# Symptoms of ocular surface disease in construction workers: comparative study with office workers

**DOI:** 10.1186/s12886-020-01548-0

**Published:** 2020-07-09

**Authors:** Sergio Hernandez-Llamas, Ana Karen Paz-Ramos, Patricio Marcos-Gonzalez, Francisco Amparo, Manuel Garza-Leon

**Affiliations:** 1 Department of Clinical Sciences, Division of Health Sciences, University of Monterrey, San Pedro Garza García, Nuevo León Mexico; 2 Cornea Service, Mass Eye, and Ear, Department of Ophthalmology, Harvard Medical School, Boston, MA USA

**Keywords:** Dry eye symptoms, OSDI, Occupational risk, office, construction workers

## Abstract

**Background:**

To investigate and contrast the prevalence of dry eye symptoms in construction workers and office workers using the OSDI questionnaire.

**Methods:**

A cross-sectional, observational study was conducted using the OSDI questionnaire to evaluate dry eye symptoms and associated risk factors. Sampled size calculation with a power of 80% and a 95% degree of confidence suggested the inclusion of 298 participants.

**Results:**

We studied 304 subjects (149 construction workers and 155 office workers). More than half (55%) of the participants presented dry eye symptoms (OSDI > 12). The average OSDI score was 21.30 ± 22.20 points, being lower in the group of construction workers (12.45 ± 17.50) than in-office workers (28.51 ± 22.99) (*p* <  0.001). Considering participants who had moderate and severe symptoms (23 to 100 points in OSDI), office workers presented dry eye symptoms 4.15 times more frequently than construction workers (OR 4.15, 95% CI 2.52, 6.85). Women presented statistical evidence of higher OSDI scores than men (32.47 ± 23.72 vs. 14.87 ± 18.48, respectively).

**Conclusions:**

construction workers have four times less risk of presenting dry eye symptoms than people working in the average office space. This highlights the pernicious effects on the ocular surface of the office environment, which poses a significant risk for the development or worsening of dry eye symptoms.

## Background

Symptoms of ocular dryness and irritation are common complaints among a variety of professionals and such symptoms can significantly influence productivity at the workplace [1]. The new TFOS DEWS II consensus emphasizes the importance of symptomatology for the diagnostic approach of dry eye, being the first requirement to classify a patient with the disease [2]. Questionnaires are the most common method used worldwide for diagnosis and follow-up of dry eye syndrome, due to its ease of application, possibility to evaluate large populations at a lower cost, and capacity to assess the impact of the disease in the patients’ quality of life [2]. There are more than 15 different questionnaires used for the diagnosis of DES, three of which assess the impact of the disease on quality of life (Impact of Dry Eye on Everyday Life [3], Dry Eye-Related Quality-of-Life Score [4] and Ocular Surface Disease Index (OSDI) [5]; the TFOS DEWS II consensus suggests the use of OSDI due to the high levels of adoption in the field [2].

The OSDI questionnaire [5] is widely used in DES epidemiologic studies [2] and assesses, with 12 questions, patients’ symptoms and impact on the quality of life across three domains: ocular symptoms, vision-related functions, and environmental triggers. Every question has a mark on a 0 to 4 scale, and the final score of the OSDI can range from 0 to 100. According to this, the OSDI can categorize patients as having a “normal” ocular surface (0–12 points), “mild” dry eye symptoms (13 to 22 points), “moderate” (23 to 32 points), or “severe” dry eye symptoms (33 to 100 points) [6].

A limited number of studies have investigated the impact of the working environment on the prevalence of dry eye symptoms (DES), and most of such studies have focused on traditional indoor working spaces. The OFFICAIR study analyzed employees of 167 office buildings in eight European countries and found that 34% of the surveyed participants presented dry eye symptoms [7]. In Japan, Uchino et al. found that 26–48% of workers in four different office spaces had severe dry eye symptoms [8]. In addition to prevalence studies, other studies have attempted to identify the risk factors associated with DES in office workers [9, 10]. The etiology of discomfort and other ocular symptoms in office environments is multifactorial and can be associated with environmental, occupational, and individual variables [9]. Individual factors can include gender, smoking, psychosocial stress, a sedentary lifestyle, among others. Environmental factors can include pollution, suspended particles, temperature, low humidity, exposure to video monitors [8–12]. Regarding the different occupations, an analysis of the Health and Nutrition Examination Survey in South Korea found that professionals and technicians had an increased risk of DES compared to farmworkers [13]. In India, Gupta et al. reported a prevalence of DES of 33% among tannery workers [14]. However, little has been reported about the occurrence of DES in construction workers.

We hypothesized that constant exposure to construction materials, dust, or debris increases the frequency of DES. This study aimed to investigate the frequency of dry eye symptoms in construction workers when compared with office workers using a validated Spanish-language version of the OSDI questionnaire [15].

### Hypothesis

Construction workers will have a higher rate of symptoms of dry eye compared to those consistently reported in office workers, mainly due to sustained exposure to dust, debris, irritant construction materials, and other environmental factors.

## Methods

This was a cross-sectional, observational, and descriptive study using a standardized questionnaire to evaluate the presence of symptoms of dry eye (Ocular Surface Disease Index) and the associated risk factors such as working hours, time spent per day on a computer or display, use of contact lenses, welding work and use of safety glasses for construction workers or the office working environment (open area vs. closed cubicles), and ocular or systemic diseases (defined as a previous diagnosis or treatment indicated by a health professional) for a total of 24 questions. For both groups, subjects required to be at least 18 years old, willingness to participate in the study, and at least six months in their current job position. The study was conducted between October and December 2017. Approval of the authorities and the Ethics Committee of the Universidad de Monterrey (UDEM) and adhered to the principles of the Declaration of Helsinki. After explaining the study, verbally informed consent was obtained from all participants, this type of consent was approved by the Ethics Committee of the University of Monterrey because the limited reading and writing capacity among many of the participants of the study (especially construction workers), the need to perform informed consent in the working place under less-than-ideal circumstances and during work hours, which also limited considerably the time available to perform both, the informed consent and the interview. The non-interventional, low-risk nature of the study and no confidential personal data were collected from the participants.

### Sample size

The target population was UDEM rectory office and construction workers of a new campus module. The construction site is located within the university campus in an urban area and next to highly trafficked avenues.

### Sampling

A simple random sampling was used, so each person remaining in the population had the same probability of being selected for the sample. Construction Workers from the foundations digging were asked to participate during working hours (8:00 am to 6:00 pm) where an investigator visited them. Office workers were visited in their workspace during working hours by an investigator. Any subject who matched the selection criteria and was willing to participate was included in the sample. The investigation was explained, and informed consent was signed before the application of the questionnaire. Office and construction workers answered the OSDI questionnaires by self-application supervised by the same investigator in all cases. The investigator explained any unknown term. Investigators did not intervene in the subject’s answers. After administration of the OSDI questionnaire, the associated demographics data and risk factors were recorded.

### Sample’s size calculation

The sample size was calculated with a comparison formula of independent proportions and in accord with the proportions described by the OFFICAIR study [7] with an expected rate of an event of 34% for office workers and 50% for construction workers, and a power of 80% and a 95% degree of confidence. The results obtained suggested the inclusion of 298 participants divided into two groups of 149 each.

### Statistical analysis

We determined the prevalence of the presence of symptoms of dry eye and the relationship between the risk factors investigated and the OSDI scores in both groups. The following factors were considered: 1) gender, 2) smoking, 3) use of contact lenses (CL), 4) hours of computer use, 5), ocular disease, and 6) systemic disease.

Odds ratios (OR) and their 95% confidence intervals (95% CIs) were calculated to measure the association between DES and risk factors. For OR analysis, we considered participants who had moderate and severe symptoms (23 to 100 points in OSDI) as individuals with the unequivocal presence of ocular surface disease (OSD) symptoms [6].

The prevalence of DES was calculated and its associations with gender, smoking, number of working hours, use of contact lenses, hours of computer use, the use of eye drops, and systemic and ocular diseases were evaluated using a logistic regression bivariate and multivariate analysis that compensates for the interactions and possible influences among the studied variables. The X2 analysis, a Student t-test, and one-way ANOVA with the post hoc Scheffe adjustment were used to analyze normally distributed data. The Mann-Whitney test was used for data that did not fit a normal distribution. Statistical significance was considered at the *P* <  0.05 level. Data analyses were carried using the statistics package SPSS V.21 (IBM Corp, Armonk, NY).

## Results

A total of 304 individuals were studied, 51% dedicated to office work and 49% to construction work, with a mean age of 34.04 ± 10.63 years and no significant differences in age between the two groups. There were 193 male (63.5%) and 111 female participants (*p* <  0.0001 ^). All the demographic characteristics are shown in Table 1.

**Table 1:** Demographic characteristics and risk factors

Variable	Total *n* = 304 (100%)	Construction workers *n* = 149 (49%)	Office workers *n* = 155 (51%)	*P* (95% CI)
Age (SD) years	34.04 ± 10.63	35.12 ± 11.40	33.03 ± 9.77	0.08 ^a^ (−0.32,4.50)
Gender M/F (%)	193/111 (63.5/36.5)	147/2 (98.7/1.3)	46/109 (29.7/70.3)	< 0.0001 ^b^
Smokers (%)	94 (30.9)	66 (44.3)	28 (18.1)	< 0.0001 ^b^
Contact lens users (%)	25 (8.2)	2 (1.3)	23 (14.8)	0.0001 ^b^
Computer hours per day	4.39 ± 4.24	0.40 ± 1.41	8.19 ± 1.86	< 0.0001 ^c^
Working hours per day	9.38 ± 1.90	9.63 ± 2.03	8.56 ± 1.02	0.0001 ^a^ (0.48,1.69)
Ocular disease	48 (15.8)	15 (10.1)	33 (21.3)	0.007 ^b^
Systemic disease	21 (6.9)	9 (6)	12 (7.7)	0.55 ^b^

^a^ Student t-test, ^b^ X2 test, ^c^ Mann-Whitney test

The mean OSDI score was 21.30 ± 22.20 units, the group of construction workers group (12.45 ± 17.50) presented significantly lower mean DES than the group of office workers (OSDI (12.45 ± 17.50 and 28.51 ± 22.99, respectively) (*p* = < 0.001) (Table 2). When comparing only male participants, the difference in OSDI scores between construction workers (12.60 ± 17.44) and office workers (22.12 ± 20.01) remained statistically significant (*p* = 0.02, 95% CI -15.53, − 3.48). Among all workers, women had statistically significant higher DES than men (OSDI 32.47 ± 23.72 vs. 14.87 ± 18.48, respectively; *p* <  0.001; 95% CI, − 12.43,22.75).

**Table 2:** Ocular surface disease symptoms’ distribution

Variable	Age	Total *n* = 304 (100)	Construction workers *n* = 149 (49)	Office workers *n* = 155 (51)	*p* (CI 95%)
OSDI		21.30 ± 22.20	12.45 ± 17.50	28.51 ± 22.99	0.001 ^a^ (−21.99,-12.75)
Symptoms free (%)	31.55 ± 9.02	139 (45.9)	96 (64.4)	43 (27.7)	< 0.001 ^b^
Mild symptoms (%)	35.17 ± 11.78	48 (15.8)	20 (13.4)	28 (18.1)	< 0.001 ^b^
Moderate symptoms(%)	41.60 ± 10.28	35 (11.5)	14 (9.4)	21 (13.5)	< 0.001^b^
Severe symptoms (%)	34.33 ± 11.06	82 (26.9)	19 (12.8)	63 (40.6)	< 0.001 ^b^

^a^ Student t-test, ^b^ X2 test

More than half of the participants (55%) had mild symptoms of dry eye (OSDI between 12 and 23). Participants who presented moderate DES were older than participants without DES (p <  0.001), mild DES (*p* = 0.04) or severe DES (*p* <  0.01). The groups and general layout are shown in Table 2.

Contact lens users presented more severe DES than non-CL users (35.25 ± 22.46 vs. 20.05 ± 21.78, respectively) with a statistically significant difference between the two groups (*p* <  0.001, 95% CI -24.17, − 6.23). Smokers presented lower DES than nonsmokers (17.56 ± 19.93 vs. 22.97 ± 22.99; *p* = 0.04 [95% CI -0.01, − 10.80]) but this disappeared when the two groups were analyzed separately.

Ocular conditions different from ametropia were present in 18 subjects, four patients had a diagnosis of dry eye and used artificial tears, three of pterygium or pinguecula, two of allergic conjunctivitis that did not require treatment, three participants had a diagnosis of glaucoma in treatment with prostaglandin analogs, and three had a diagnosis of cataract. Systemic diseases were present in 19 subjects, seven with diabetes mellitus type 2, five with allergies or asthma, and three with high blood pressure. One participant was taking oral contraceptives.

Due to the nature of their work, participants in the construction group were more often men and had less exposure to computer work. Additionally, they referred to less contact lens use and smoking more often than office workers (Table 1). The average working time during a day was 9.38 ± 1.90 h, being longer in construction workers (9.63 ± 2.03) than in-office workers (8.56 ± 1.02) (*p* <  0.001).

Ninety-five office employees worked within a closed office and had a significantly less DES than employees working in open areas (cubicles) (OSDI 26.52 ± 21.37 vs. 35.02 ± 24.72) (*p* = 0.03, 95% CI -16.17, − 0.82). Demographic characteristics and risk factors are shown in Table 3.

**Table 3:** Office workers’ characteristics

Variable	Total *n* = 155 (100%)	Closed office *n* = 95 (61.3%)	Open area *n* = 60 (38.7%)	*p* (CI 95%)
Age (SD) years	33.03 ± 9.77	32.27 ± 9.13	34.22 ± 10.66	0.22 ^a^ (−1.2,5.1)
Gender M/F (%)	46/109 (29.7/70.3)	31/64 (32.6/67.4)	15/45 (25/75)	0.31 ^b^
Smokers (%)	28 (18.1)	23 (24.2)	5 (8.3)	0.01^b^
Contact lens users (%)	23 (14.8)	15 (15.8)	8 (13.3)	0.67 ^b^
Computer hours per day	8.19 ± 1.86	8.24 ± 1.79	8.20 ± 1.81	0.87 ^c^
Working hours per day	8.56 ± 1.02	8.47 ± 1.60	8.53 ± 1.26	0.80 ^a^ (−0.4,0.5)
Ocular disease	33 (21.3)	15 (15.8)	18 (30)	0.03 ^b^
Systemic disease	12 (7.7)	7 (7.4)	5 (8.3)	0.82 ^b^

^a^ Student t-test, ^b^ X2 test, ^c^ Mann-Whitney test

**Fig. 1:**
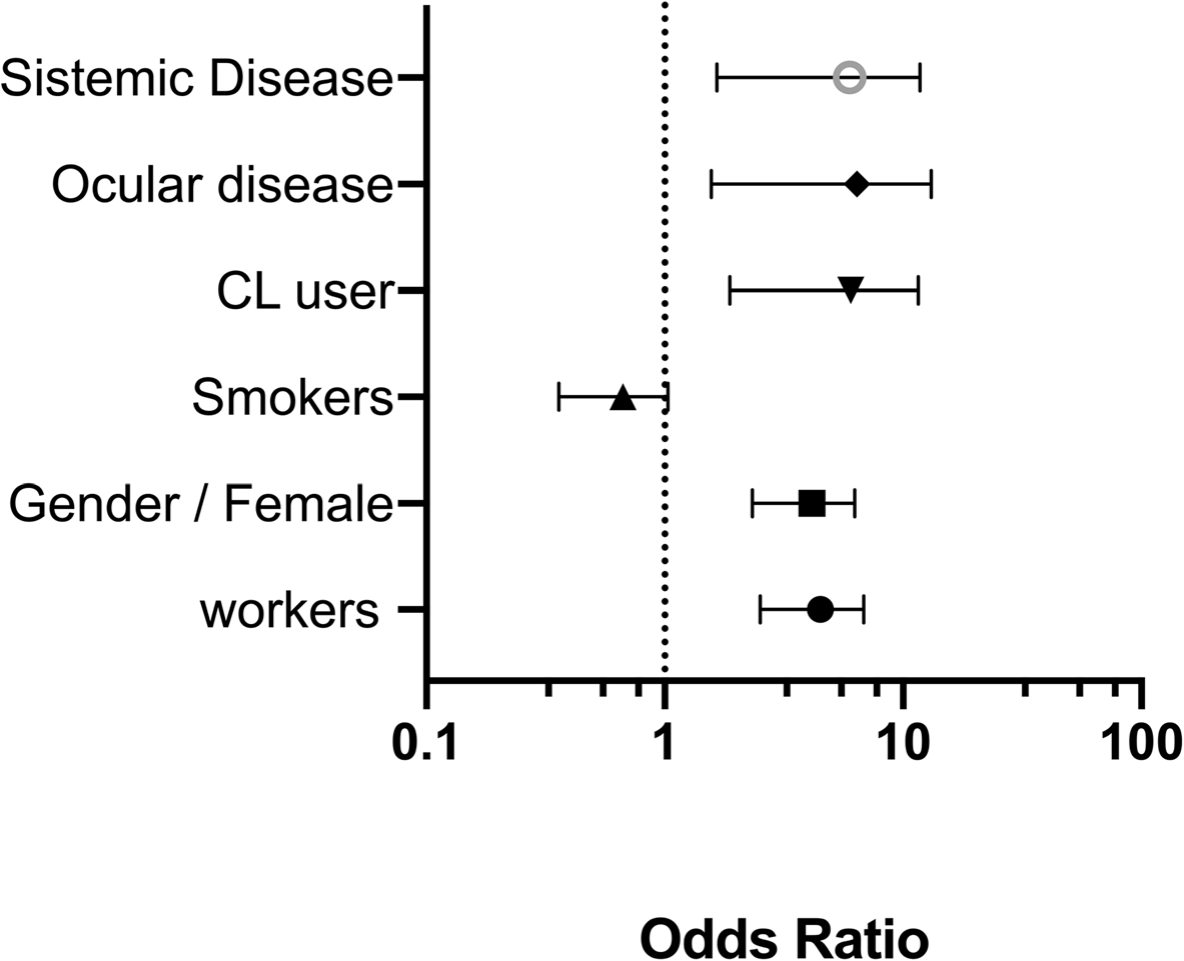
OR Univariate DES risk analysis

Office workers presented DES 4.15 times more often than construction workers (OR 4.15. 95% CI 2.52, 6.85), a result that also maintained when only males were compared (OR 2.43, CI 95% 1.20, 4.91). When evaluated separately, women presented DES 3.82 times more often than men (OR 3.82, 95% CI 2.48, 5.91). Among office workers, women presented DES more often than men (OR 2.09, 95% CI 1.04, 4.23). CL users had 4.67 times more risk of presenting DES than those that did not wear CL (95% CI 1.88, 11.57). (Fig. 1).

**Fig. 2:**
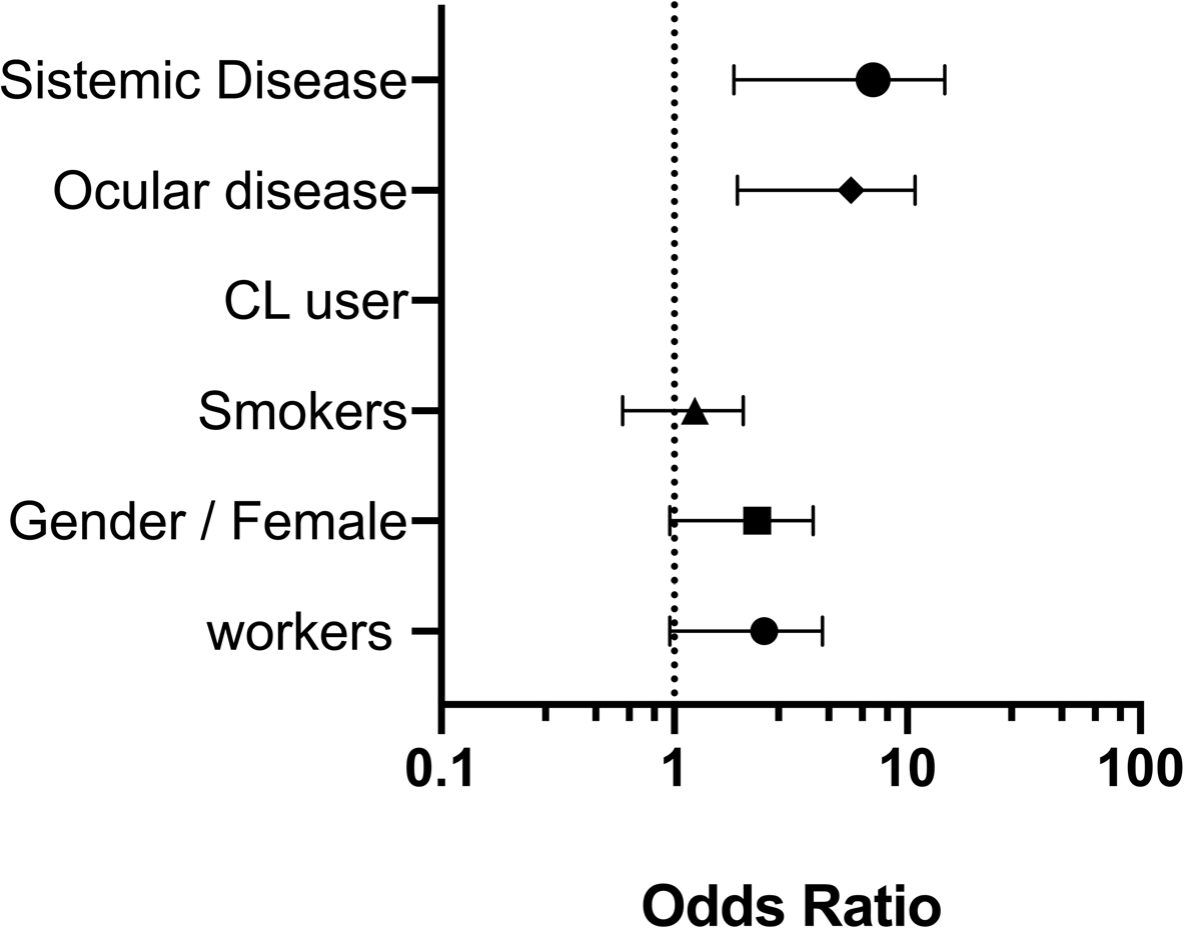
OR multivariate DES risk analysis after adjusting for a working group, gender, smoking habit, CL use, and ocular and systemic disease

Figure 2 shows the multivariate DES risk analysis after adjusting for a working group, gender, smoking habit, CL use, and ocular and systemic disease. The mean relative humidity during the study days was 40.3 ± 1.37% (range 38 to 43%) in the office building and 59.75 ± 2.62% (range 54 to 63%) in the outdoor space where the construction site was located (*p* <  0.01).

## Discussion

In this study, we evaluated the impact of environmental factors on the presence of dry eye symptoms in construction workers when compared with office workers. We found that office workers present more severe symptoms than construction workers, and the risk of suffering from DES persisted after adjusting for all the studied variables. Different factors can influence these results, but, perhaps, mainly by the office workers’ exposure to sustained computer use in significantly drier environments since in our study, we found that the office environment was 50% drier than air in the outdoor space. A report from Kawashima et al. supports these results, they showed that DES are commonly reported by personal using video screens [12]. Similarly, Uchino et al. reported an increased risk of DES when video screens are used for more than 4 hours [8]. Lee et al. studied 6023 participants and showed that besides computer use, other environmental factors could influence the development of DES. In their study, 16% of the participants presented dry eye symptoms, with professionals, managers, legislators, and senior officers presenting more risk of DES than factory workers. Also, he described that people who work outdoors have a lower risk of dry eye symptoms [13].

In accord with many other reports [8, 13, 15–22], in the present study, women presented higher scores OSDI scores than men; thus, gender differences may have influenced differences between the two groups since there was a higher ratio of women among office workers. However, after correction for gender in the multivariate analyses, statistically significant differences linked to gender disappeared.

Contact lens use has been associated with DES [15, 18, 20, 23, 24], in our study, we found a higher score on the OSDI questionnaire in CL users compared with nonusers. However, when performing multivariate analysis, this influence lower the OR, probably due to the presence of more CL users among office workers [23] than among construction workers.

In this study, a previous diagnosis of ocular or systemic disease was associated with an increased risk to present DES. Previous studies have shown a high prevalence of ocular DES in patients attended by the ophthalmologist [25, 26], in addition to the ones with specific diseases such as glaucoma [8], diabetic retinopathy [27] and ocular surface diseases [28, 29]. The relationship between DES and systemic diseases has been extensively studied, Paulsen et al. showed that various systemic diseases such as allergies, arthritis, thyroid disease and the use of drugs such as systemic antihistamines and steroids are related to the presence of dry eye [24].

Smoking has been reported to be a risk factor for DES [22, 30]. Wolkoff reported that cigarette smoke affects the pre-corneal tear film and the break-up time [23]. Lee et al. reported a higher prevalence of dry eye symptoms in smokers [13]. De Kluizenaar et al. [7] and Ranciere et al. [31] documented that tobacco smoke has been associated with a higher prevalence of DES. In this study, smokers presented lower OSDI scores, and the multivariate analysis ultimately revealed no real associations between smoking and OSDI scores. The OSDI score differences between smokers and non-smokers, most smokers were part of the group with the lower OSDI scores (construction workers). In comparison, non-smokers were mostly office workers (the group with higher OSDI scores), hence, as suggested by the multivariate analysis, we speculate that the workers’ occupational exposure influenced these results.

Due to the nature of the selected and studied occupations, the population was heterogeneous regarding gender, and this situation may have induced some artifacts and limitations to this study. This study did not include a clinical evaluation of the ocular surface, and thus, all that was measured were symptoms of dry eye and not ocular surface or dry eye disease. Based on results observed with some variables, such as the smoking habit and CL use, the sample size is limited to draw firmer conclusions.

The findings of this study underline the serious need to inform the population working in offices, the largest workforce in western countries, on the risks of developing dry eye symptoms, and provide advice on measures to minimize them. These measures should include avoiding long uninterrupted periods of computer work, regular breaks to allow for regular blinking and ciliary relaxation, and to demand better humidity controls in the workplace.

## Conclusion

Despite the multiple adverse environmental working conditions, construction workers have four times less risk of presenting dry eye symptoms than people working in the average office space. This highlights the pernicious effects on the ocular surface of the office environment, which poses a significant risk for the development or worsening of dry eye symptoms.

## Data Availability

The datasets used and/or analyzed during the current study are available from the corresponding author on reasonable request.

## Abbreviations

DE: Dry eye disease
DES: Dry eye symptoms
CL: Contact lenses
OSDI: ocular surface disease index questionnaire
UDEM: University of Monterrey
OR: Odds ratio
OSD: ocular surface disease
95% CI: 95% confidence interval
